# Therapeutic Mechanism of Kynurenine, a Metabolite of Probiotics, on Atopic Dermatitis in Mice

**DOI:** 10.3390/foods14101816

**Published:** 2025-05-20

**Authors:** Yixuan Li, Mingxin Li, Qingyu Ren, Chunqing Ai, Shugang Li, Huan Li, Shouhao Zhao, Donglin Sui, Xiaomeng Ren

**Affiliations:** State Key Laboratory of Marine Food Processing and Safety Control, National Engineering Research Center of Seafood, School of Food Science and Technology, Dalian Polytechnic University, Dalian 116034, China; lyxqaq9527@163.com (Y.L.); 15998327512@163.com (M.L.); 19841133861@163.com (Q.R.); acqdongying@163.com (C.A.); lishugang688@163.com (S.L.); lh276704997@163.com (H.L.); shouhao1105@163.com (S.Z.); sdl3303@163.com (D.S.)

**Keywords:** gut microbiota, oxidant stress, tryptophan metabolism

## Abstract

Atopic Dermatitis (AD) is a common inflammatory skin disease characterized primarily by its chronic and recurrent nature. This has a significant impact on productivity and human longevity. Dysbiosis of gut flora has been demonstrated to be significantly associated with the progression of AD. In our previous research, it was shown that *Lactobacillus rhamnosus* RL5-H3-005 (RL) and *Pediococcus acidilactici* RP-H3-006 (RP) have the ability to reduce the risk of disease in AD mice through the gut–mammary axis. Based on our previous work, this study aims to further investigate the effects of kynurenine (KYN), a metabolite of RL and RP, on AD mice induced by 2, 4-dinitrofluorobenzene (DNFB). In this study, we found that supplementing KYN in AD mice effectively alleviates the pathological symptoms of atopic dermatitis and further improves the levels of SCFAs in their intestines. Further research indicates that KYN’s therapeutic effects on AD are primarily manifested in the reduction of secretory immunoglobulin A (sIgA), immunoglobulin E (IgE), interleukin-4 (IL-4), IL-5, IL-13, and thymic stromal lymphopoietin (TSLP) levels in mice, while also repairing the intestinal barrier function of AD mice. Overall, the metabolites KYN of probiotics RL and RP can regulate the levels of SCFAs of mice, potentially improving the symptoms of AD mice through the gut–skin axis.

## 1. Introduction

Atopic dermatitis (AD) represents a significant global health challenge, which is characterized by dry skin, eczema-like rashes, intense itching, redness, and scaling [1]. The disease is characterized by a chronic course and high recurrence rate, severely impacting the productivity and quality of life of patients [2]. Clinical data indicate that the prevalence of AD in infants and young children ranges from 10% to 15%, placing a substantial burden on the affected children and their families [3]. As our understanding of the gut–skin axis expands, an increasing number of studies are exploring the potential for alleviating AD via modulation of the gut microbiota [4,5]. The gut–skin axis plays an important role in modulating immune responses, enhancing nutrient absorption, and influencing hormonal balance [6]. Furthermore, the intestinal barrier integrity, relies on the expression of tight junction proteins, is crucial for maintaining intestinal homeostasis, and its disruption triggers immune responses, exacerbating AD [7,8].

The immune-inflammatory response is the primary driver of the pathogenesis of AD [9,10]. In contrast to a healthy state, the balance between T helper 1 (Th1) and Th2 immune responses is disrupted in AD patients, accompanied by an imbalance in their immune regulatory factors. They promote the secretion of alarm proteins, such as thymic stromal lymphopoietin (TSLP), when allergens penetrate the skin barrier, thereby activating dendritic cells and innate lymphoid cells [11,12,13]. The activation of dendritic cells and innate lymphoid cells induces the secretion of IL-5 and IL-13, which in turn activates Th2 cells [14,15,16]. Th2 cell activation increases the production of cytokines (e.g., IL-4, IL-5, and IL-13), which simultaneously induces the secretion of immunoglobulin E (IgE), which exhibits a high affinity for basophil granulocytes and mast cells [17,18]. Tryptophan metabolism has been implicated in the development of various diseases [19,20]. Current studies indicate that the tryptophan metabolic pathway is involved in the treatment of Alzheimer’s disease [21], inflammatory bowel disease [22], periodontal inflammation [23], food allergies [24], and depression [25]. At present, tryptophan has become a potential, new, and natural food supplement, which has rich and pleiotropic effects on human body. Tryptophan metabolites exhibit anti-inflammatory effects, thereby enhancing the immune function of these patients [20,26]. Under a black rice diet, the levels of intestinal metabolites, e.g., indole-3-lactic acid and indole, were significantly increased in mice. These metabolites stimulate the aryl hydrocarbon receptor (AHR) pathway, inhibiting colorectal cancer CRC cell proliferation and colorectal tumorigenesis [27].

Our previous study showed that *Lactobacillus rhamnosus* (RL) and *Pediococcus acidilactici* (RP) had the potential to modulate gut microbiota and immune responses, which can improve the asthma symptoms and AD in mice models [8,28]. Moreover, both RL and RP exerted anti-inflammatory effects by regulating intestinal inflammatory infiltration, levels of pro-inflammatory factors, and oxidative stress [29]. However, the precise mechanisms by which microbial metabolites contribute to the treatment of AD remain poorly understood. To further explore the effect of microbial metabolites on AD, the effects of RL and RP on tryptophan metabolism were assessed, and a pivotal tryptophan metabolite kynurenine (KYN) was identified. To evaluate the effect of KYN on AD, KYN was administered to AD mice induced by DNFB, and biochemical indicators were measured to verify its preventive effect. Additionally, the composition of short chain fatty acids (SCFAs) and the integrity of the intestinal barrier were assessed. Consequently, we speculate that KYN have potential for mitigating AD.

## 2. Methods and Materials

### 2.1. Sources of Probiotics

RL and RP are derived from the feces of healthy infants and are patented strains of our research group. They were retrieved from the −80 °C freezer, activated and passaged, and then cultured in MRS broth medium (Hope Bio-Technology Co., Ltd., Qingdao, China) under aerobic condition at 37 °C for 24 h. The total number of colonies after probiotic culture is approximately 1 × 10^9^ CFU/mL.

### 2.2. Animal Experimentation

Female BALB/c mice (six weeks old, SPF) were provided by Changsheng Biotechnology Co., Ltd. (Changchun, China). All mice were housed in an IVC system, with the temperature maintained at 20 ± 2 °C, humidity at 50 ± 5%, and a 12-h light/dark cycle. All animal experimental procedures strictly adhered to the guidelines of the National Committee for the Management of Laboratory Animals and were approved by the Animal Ethics Committee of Dalian Polytechnic University (No. DLPU2024025). At the end of the mice experiment, blood samples were immediately obtained from eye sockets after isoflurane anesthesia. All mice were euthanized via cervical dislocation, and organs and tissues were collected immediately for further analysis.

**Figure 1:**
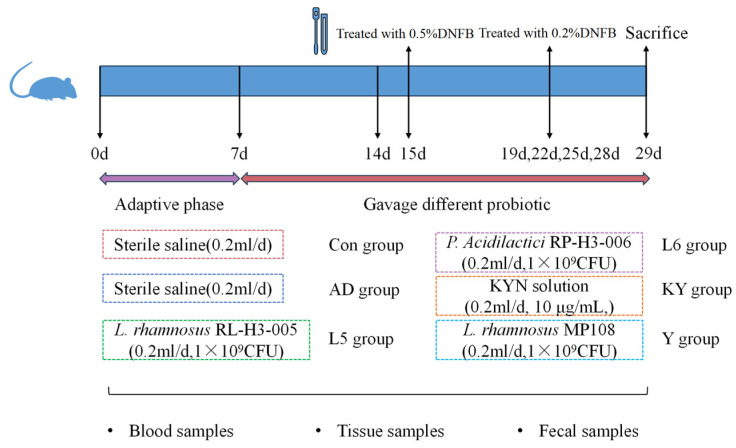
Diagram of the animal study design.

After one-week of adaptation, mice were divided into six groups (*n* = 5/group): mice that were treated solely with PBS (CON), the model group (AD), mice that were gavaged 1 × 10^9^ CFU of RL (RL), RP (RP), mice that were gavaged with 200 μL of KYN solution (10 μg/mL) (KYN), and mice that were gavaged 1 × 10^9^ CFU of *L. rhamnosus* MP108 (200 μL) (Y). After one week, except for the CON group, the other groups were treated with 0.5% DNFB on the ears, followed by additional treatments with 0.2% DNFB at four-days intervals for four times. Mice were sacrificed following the final treatment (Figure 1).

### 2.3. Thickness and Weight of Ear Tissue

The thickness of mouse ears was measured using a micrometer in the range of 0–25 mm (Mitutoyo, Shanghai, China) before killing the mice. After sacrificing the mice, a piece of ear plate with a diameter of 6 mm was excised from the mice and weighed.

### 2.4. Histological Analysis

Fixed ear tissues were embedded in wax and cut into 5 μm sections for subsequent hematoxylin and eosin (H & E) and toluidine blue staining. Jinzhiyuan Biotechnology Co., Ltd. (Shenyang, China) is responsible for staining the tissue. Tissues were visualized at 100 μm using a light microscope (Nikon Eclipse Ci, Tokyo, Japan).

### 2.5. Determination of Biochemical Index

Serum was separated by centrifugation at 1000× *g* for 20 min at 4 °C using a microcentrifuge (Thermo Fisher, Waltham, MA, USA). Ileum tissues were weighed and homogenized in ice-cold PBS at a 1:9 (*w*/*v*) ratio. The resulting homogenates were centrifuged at 600× *g*, 4 °C for 10 min, and then the levels of sIgA in the homogenates were measured using kits. Additionally, the levels of IL-4, IL-5, IL-13, IgE, and TSLP in the serum were quantified using ELISA kits (mlbio, Shanghai, China).

### 2.6. RT-PCR

Total RNA was extracted from ileum and ear tissue homogenates using Trizol reagent (Sangon, Shanghai, China). RNA concentration and purity were assessed using a NanoDrop spectrophotometer (Thermo Fisher, Waltham, MA, USA). Complementary DNA (cDNA) was synthesized using the KR118 reverse transcription kit (Tiangen, Beijing, China). Quantitative analysis of target gene expression was performed using a real-time PCR system with the SuperReal PreMix Plus Kit (Takara, Beijing, China) and gene-specific primers (Table S1). Relative quantification of target genes was calculated using the 2^−ΔΔCT^ method, with GAPDH serving as the internal control [8].

### 2.7. SCFAs Analysis

SCFAs levels in fecal samples was conducted following a previously established method. In total, 100 mg of mouse faces were treated with 50% dilute sulfuric acid followed by the addition of an internal standard (2-methylbutyric acid) and ether. After vortexing, centrifugation, and purification, the resulting extracts were subjected to GC-MS analysis with an Agilent 7890/5975 system (Agilent, Santa Clara, CA, USA) equipped with a VFWAXms capillary column. The parameters of MS were 70 eV electron ionization and a scan range of M/z 30–300 (2.5 min solvent delay). The unknown SCFA concentration was finally determined by linear regression using the generated standard curve.

### 2.8. Statistical Analysis

Statistical analyses were performed using GraphPad Prism 10. Data are expressed as the mean ± standard error of the mean (SEM). One-way ANOVA with a Tukey post hoc test was used to assess statistical significance among multiple groups (*p* > 0.05 (n.s.), * *p* < 0.05, ** *p* < 0.01, and *** *p* < 0.001). Use Student’s *t*-test to evaluate statistical significance between two groups (*p* > 0.05 (n.s.), * *p* < 0.05, ** *p* < 0.01, and *** *p* < 0.001) (n.s., not significant).

## 3. Results

### 3.1. RL and RP Influenced the Metabolites in AD Mice

**Figure 2:**
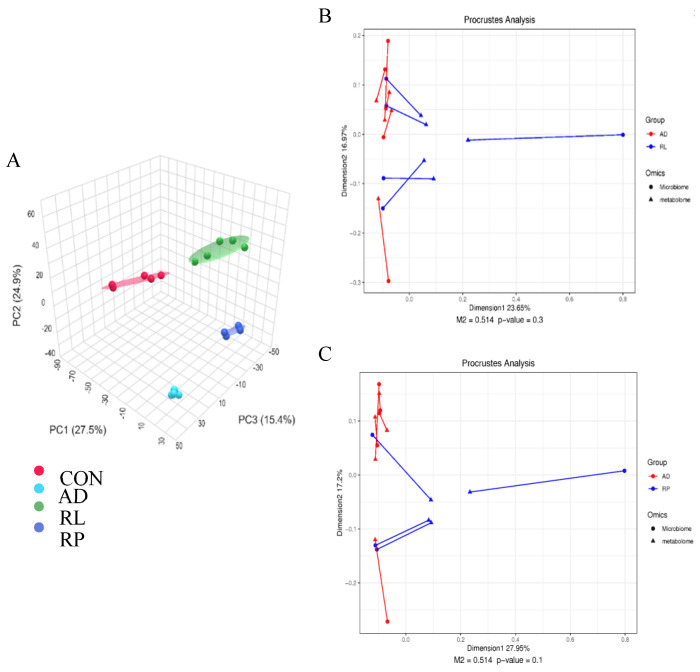
Effects of RL and RP on the microbiome and metabolism of Ad mice. (**A**) Principal Coordinate Analysis (PCoA) based on Bray–Curtis distances. Procrustes analysis integrating microbiome and metabolome data to assess the similarity between (**B**) AD vs. RL comparison (M^2^ = 0.514, *p* = 0.3) and (**C**) AD vs. RL comparison (M^2^ = 0.514, *p* = 0.1).

**Figure 3:**
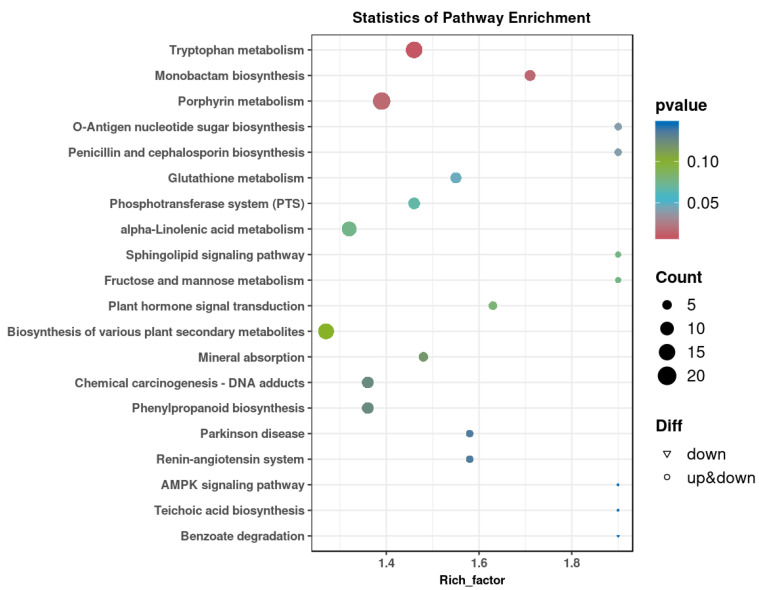
KEGG enrichment map of differential metabolites after RL treatment.

**Figure 4:**
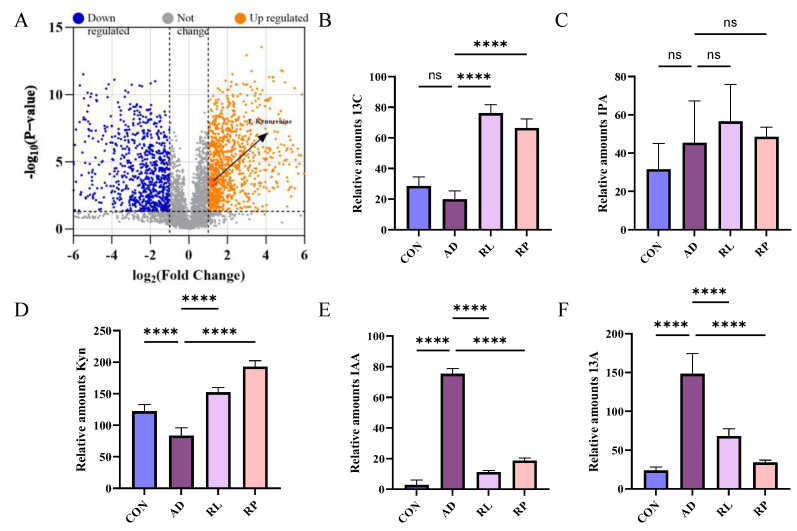
Differential metabolite analysis and pathway-specific changes among treatment group. (**A**) Volcano plot illustrating differential metabolites between the AD and RL groups. (**B**) KEGG pathway enrichment of butanoate metabolism-associated compounds. (**C**) Differential abundance of compounds involved in tryptophan metabolism. (**D**) Expression of bile acid-related metabolites. (**E**) Alteration in levels of histidine metabolism-associated compounds. (**F**) Changes in linoleic acid metabolism among groups. Each value was expressed as mean ± SEM (*n* = 5). ns = no significance, **** *p* < 0.0001 vs. AD group.

As shown in Figure 2A, significant separations in the microbial communities were observed among the CON, AD, RL, and RP groups. The microbiota treated with RL were most similar to the CON group, while both the RL and RP treatment groups differed from the AD group, showing no significant consistency. Procrustes analysis integrating microbiome and metabolome data was used to assess the similarity between the two omics layers. Each pair of connected points represents a sample’s microbiome (black) and metabolome (grey) profiles. In both comparisons, there was no significant consistency, indicating that after RL and RP treatments, both the microbiota and metabolism underwent significant changes compared to those in the AD-state mice (Figure 2B,C). Through heatmap analysis, significant differences in tryptophan metabolism were observed after RL treatment (Figure 3). The volcano plot revealed that the treatment group significantly upregulated the metabolite KYN, which is associated with inflammation and immune regulation (Figure 4A). The relative abundance of selected metabolites or pathway-related compounds across different groups revealed that the treatment group exhibited significant differences from the AD group in metabolites associated with gut health, anti-inflammatory function, and immune function (Figure 4B–F) [30,31]. All of the above demonstrates that KYN plays a significant role in the treatment of AD in mice. Based on this, we will continue to explore the therapeutic mechanisms of KYN in AD mice in subsequent research.

### 3.2. KYN Ameliorated the Pathological Symptoms in AD Mice

**Figure 5:**
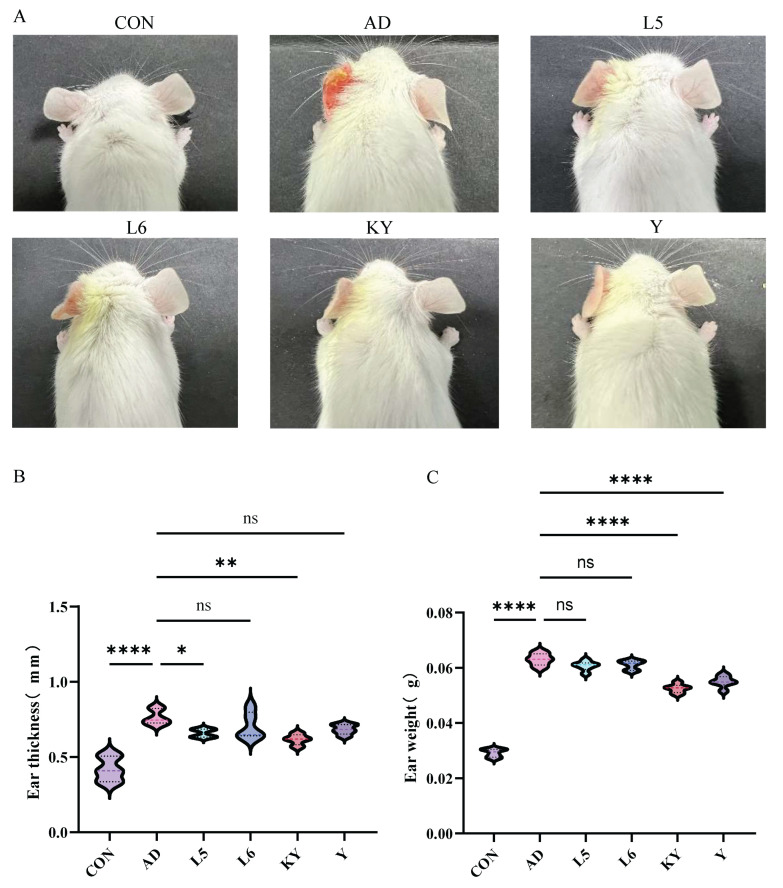
Improvement of the pathological state of AD mice by Kyn. (**A**) The external appearance of the mice ear. (**B**) Mice ear thickness. (**C**) Mice ear weight. Each value was expressed as mean ± SEM (*n* = 5). ns = no significance, * *p* < 0.05; ** *p* < 0.01, and **** *p* < 0.0001 vs. AD group.

**Figure 6:**
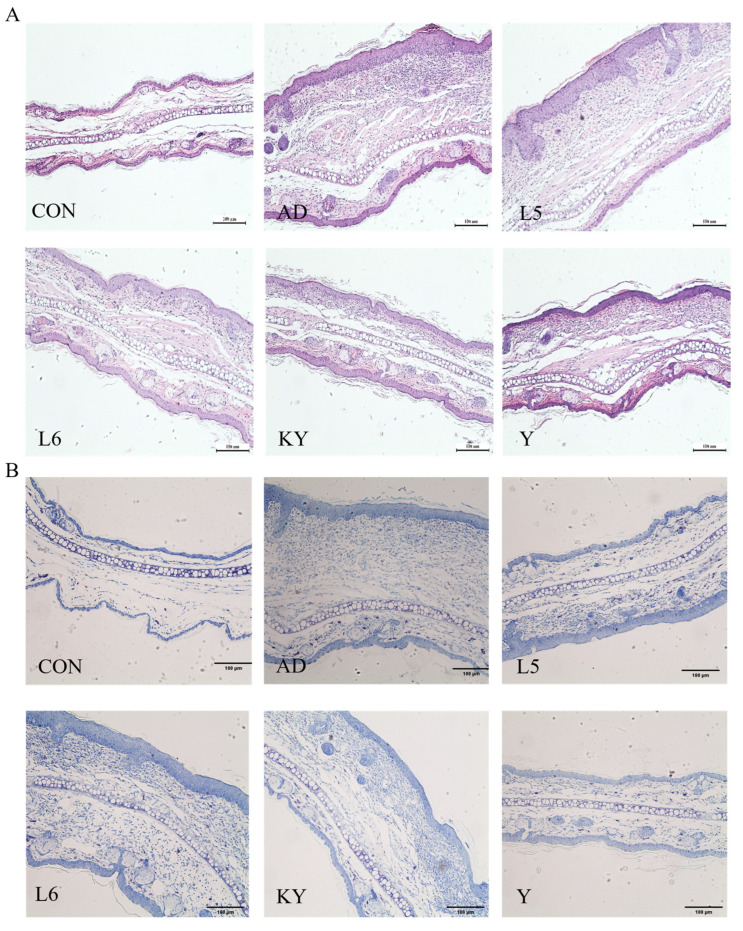
Improvement of the pathological state of AD mice by KYN. (**A**,**B**) Representative images of ear tissue stained with H&E and toluidine blue; scale bar = 100 μm.

Mice in the CON group exhibited normal tissue architecture, whereas those in the AD group displayed pronounced ear thickening, erythema, scabbing, and severe tissue contraction (Figure 5A). In contrast, KYN treatment visibly alleviated ear edema and hyperemia. Histopathological examination revealed extensive inflammatory cell infiltration, irregular epidermal hyperplasia, and pronounced dermal keratinization in the AD group. Treatment with RL and RP significantly attenuated DNFB-induced dermal and epidermal thickening and markedly reduced inflammatory cell infiltration (Figure 6A). Furthermore, ear thickness and weight were significantly decreased in the KYN-treated group (Figure 5B,C), indicating that both KYN and the probiotic strains RL and RP effectively mitigate the pathological features associated with AD in mice.

### 3.3. KYN Regulates SlgA and Cytokine Levels

**Figure 7:**
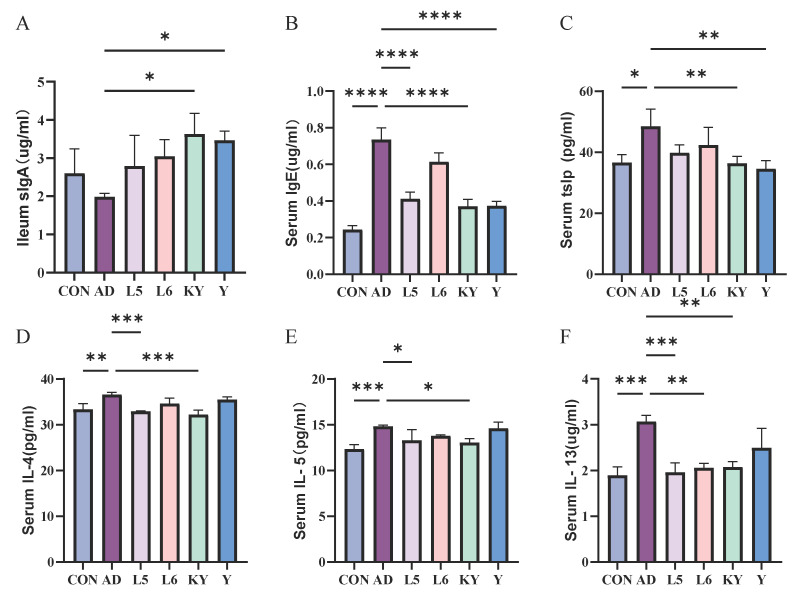
The effect of KYN on the level of sIgA in the ileum (**A**) and IgE (**B**), TSLP (**C**), IL-4 (**D**), IL-5 (**E**), and IL-13 (**F**) in the serum. Each value was expressed as mean ± SEM (*n* = 5). * *p* < 0.05; ** *p* < 0.01; *** *p* < 0.001; and **** *p* < 0.0001 vs. AD group.

SlgA is a crucial antibody in the human body, playing a significant role in intestinal immunity. Through experimental data analysis, we discovered that there was no difference in sIgA levels in the ileum of mice between the CON group and the AD group. However, both the KY and Y treatment groups significantly increased the sIgA content in the ileum of AD mice (Figure 7A). In the determination of serum biochemical indicators, compared to the control group, supplementation with KYN resulted in a significant decrease in the levels of lgE and TSLP, as well as a notable reduction in the levels of pro-inflammatory cytokines IL-4, IL-5, and IL-13 in the serum (Figure 7B–F).

### 3.4. KYN Ameliorated the Intestinal Barrier Function in AD Mice

**Figure 8:**
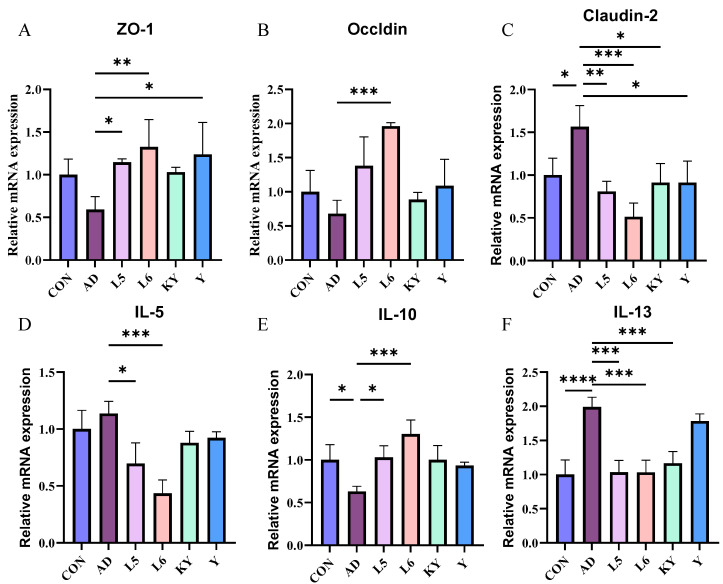
Effect of KYN on intestinal barrier and inflammatory response. The mRNA levels of ZO-1 (**A**), Occludin (**B**), and Cauldin-2 (**C**) in the ileum tissues, and the levels of IL-5 (**D**), IL-10 (**E**), and IL-13 (**F**) in the ear tissues. Each value was expressed as mean ± SEM (*n* = 5). * *p* < 0.05; ** *p* < 0.01; *** *p* < 0.001; and **** *p* < 0.0001 vs. AD group.

The integrity of the intestinal barrier largely influences an individual’s health, so we measured the mRNA expression of ZO-1, Occludin, and Claudin-2 in mice (Figure 8A–C). Compared with the CON group, the levels of Zo-1 and Occludin in the AD group did not differ significantly, while the level of Claudin-2 increased significantly. Compared with the AD group, L 5 and L 6 increased the expression of ZO-1 and decreased the level of Claudin-2. KYN significantly decreased the expression of Claudin-2 and slightly increased the expression of Zo-1 and Occludin. It indicates that KYN can improve the intestinal barrier function to a certain extent, which helps to pass through the intestine and then affect the subsequent function.

### 3.5. KYN Suppressed Aberrant Immune Response in the Ear Tissues

At the mRNA level, the IL-13 mRNA expression in the AD group was significantly elevated compared to the control group, whereas no significant differences were observed in IL-5 and IL-10. Conversely, treatment with KYN reduced the IL-13 levels in AD mice without significantly affecting IL-5 and IL-10 (Figure 8D–F). This demonstrates that KYN has a significant effect in terms of anti-inflammatory action.

### 3.6. KYN Elevate SCFAs Levels in AD Mice

**Figure 9:**
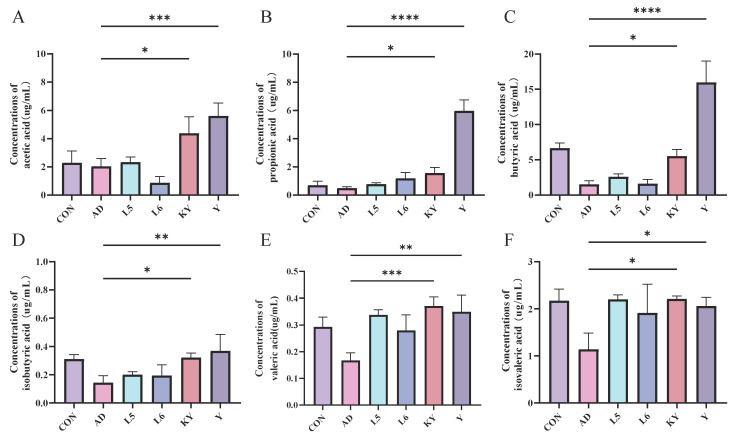
KYN improves short-chain fatty acids (SCFAs) levels in AD mice. Concentrations of (**A**) acetic acid, (**B**) propionic acid, (**C**) butyric acid, (**D**) isobutyric acid, (**E**) valeric acid, and (**F**) isovaleric acid. Each value was expressed as mean ± SEM (*n* = 5). * *p* < 0.05; ** *p* < 0.01; *** *p* < 0.001; and **** *p* < 0.0001 vs. AD group.

As shown in the figure, the concentration of six types of short-chain fatty acids in the cecum of the DSS group mice showed a decreasing trend compared to the CON group (Figure 9). After administration in different treatment groups, the levels of short-chain fatty acids increased, particularly in the KYN group and the Y group, with the most significant increase in pentanoic acid observed after KYN treatment. The results indicate that KYN can alleviate colitis by regulating the levels of short-chain fatty acids.

## 4. Discussion

AD is a common chronic inflammatory skin disorder. Current standard treatments primarily involve corticosteroids, immunosuppressants, and other pharmacological agents [32,33,34]. However, these therapies often fail to achieve sustained remission. Prolonged use is frequently associated with drug dependency, increased healthcare costs, and adverse side effects. As a multifactorial condition, AD is influenced by various environmental factors and is characterized by dysbiosis not only of the gut microbiota but also of the cutaneous microbial community [35,36,37,38]. Clinical data indicate that the tryptophan metabolic pathway contributes to alleviating symptoms of AD [39]. This study explores the therapeutic potential of kynurenine (KYN), a metabolite identified from RL and RP metabolic profiles, in mitigating symptoms of atopic dermatitis (AD) in mice. The findings demonstrate that KYN exhibits immunomodulatory properties and contributes to the restoration of intestinal barrier function. In AD-like mice, impaired skin barrier integrity and heightened inflammatory cell infiltration are observed, driven by dysregulated immune responses. As a result, AD is characterized by pronounced skin inflammation and exaggerated Th2-mediated immune activity during the acute phase [40].

Our results are well aligned with and extend previous findings on the role of the gut microbiota and its metabolites in immune-mediated skin disorders. The concept of the gut–skin axis, a bidirectional communication network linking gut microbiota and skin homeostasis, is increasingly recognized in dermatological research [36,38]. Dysbiosis in gut microbiota composition has been implicated in the initiation and exacerbation of AD, where reduced microbial diversity and the depletion of beneficial commensals contribute to systemic inflammation and skin barrier dysfunction [41,42,43]. Importantly, the tryptophan metabolic pathway has emerged as a critical immunometabolic axis in chronic inflammatory diseases. Tryptophan, an essential amino acid, can be metabolized into several bioactive compounds, including kynurenine, indole derivatives, and serotonin [22,44]. Among these, kynurenine has been shown to suppress pro-inflammatory responses and promote immune tolerance via the activation of the aryl hydrocarbon receptor (AhR), the modulation of regulatory T cell populations, and the inhibition of effector T cell activation [45,46,47]. Our findings echo this body of work and demonstrate that exogenous KYN can recapitulate many of the beneficial effects attributed to probiotic supplementation, suggesting that microbial metabolites may be central effectors of host–microbiota interactions. Furthermore, the immunological results—particularly the suppression of IL-4, IL-5, IL-13, TSLP, and IgE—indicate that KYN effectively mitigates the Th2-skewed immune response, which is a hallmark of AD pathophysiology. Notably, increased levels of IL-10 in KYN-treated mice support the notion that KYN may promote a more tolerogenic immune environment, possibly via the enhancement of regulatory T cells or AhR-mediated pathways. These findings are consistent with previous observations in allergic asthma and food allergy models, where tryptophan metabolites reduced disease severity and inflammatory cytokine production. In addition to its immunoregulatory effects, KYN also exerted a notable impact on intestinal barrier integrity, as reflected by increased mRNA levels of ZO-1 and Occludin and decreased Claudin-2 expression in the ileum. These proteins are essential components of the tight junction complex and are responsible for maintaining the selective permeability of the intestinal epithelium. The disruption of these junctions permits the translocation of microbial products into systemic circulation, triggering systemic inflammation and exacerbating AD symptoms. Our findings suggest that KYN reinforces gut barrier function, thereby limiting systemic immune activation and contributing to symptom relief in AD. The elevated SCFA levels observed in KYN-treated mice provide further mechanistic insight into its therapeutic efficacy. SCFAs, particularly butyrate, propionate, and acetate, are key microbial metabolites known to modulate host immunity, maintain epithelial barrier function, and suppress inflammatory signaling [47,48,49]. Butyrate, for example, has been shown to enhance tight junction protein expression, increase mucin production, and induce Treg differentiation. By promoting SCFA production, KYN may indirectly support a gut microenvironment conducive to immune homeostasis and barrier protection. This synergistic effect between microbial and metabolic pathways underscores the systemic nature of KYN’s actions and highlights the potential for metabolic reprogramming in treating inflammatory diseases.

While our study offers valuable insights, several limitations must be acknowledged. First, although KYN administration effectively ameliorated AD symptoms in a murine model, the translation of these findings to human subjects remains speculative. Human clinical trials are essential to confirm safety, dosing, and efficacy. Second, the mechanism of action underlying KYN’s effects remains incompletely understood. While Aryl Hydrocarbon Receptor activation is a plausible pathway, further experiments involving receptor antagonists, gene knockouts, or transcriptomic profiling are needed to confirm the exact molecular targets. Third, the study focused on a single microbial metabolite, whereas probiotic strains produce a broad spectrum of metabolites that may act synergistically. It is possible that other co-metabolites derived from RL and RP contribute to the observed therapeutic benefits. Lastly, the impact of KYN on the skin microbiota, another key player in the gut–skin axis, was not assessed in this study and warrants future investigation. This research supports the growing paradigm that microbial metabolites act as key mediators in host–microbe interactions and have far-reaching effects on distant organs through systemic circulation. The identification of KYN as a potent postbiotic agent capable of mitigating AD symptoms opens up exciting possibilities for microbiota-targeted therapies in dermatology. Compared to live probiotic administration, postbiotic approaches offer greater stability, lower risk of translocation, and the potential for precision medicine by selecting specific metabolites based on individual host or microbiome profiles. Future studies should further investigate the translational potential of these findings by evaluating the effects of KYN in human subjects with AD and examining its interactions with host genetics, diet, and microbiome composition. Additionally, co-administration strategies involving KYN and SCFA precursors or synergistic probiotics may yield enhanced therapeutic efficacy. Systems biology approaches, including integrated multi-omics analyses and machine learning models, could further elucidate the complex interactions between microbial metabolites, immune signaling, and skin health.

## 5. Conclusions

This study demonstrates that KYN, a metabolite of RL and RP, possesses the potential to modulate immune responses and short-chain fatty acids, thereby contributing to the amelioration of AD. It provides preliminary evidence that KYN may exert preventive effects on AD through the regulation of short-chain fatty acids. However, further mechanistic studies need to be continued. The mechanism of KYN through the gut–skin axis warrants further investigation, including its impact on the gut microbiota, the relationship between microbial metabolites and the gut microbiota, and whether KYN induces specific changes in the skin microbiota of AD patients.

## Data Availability

The original contributions presented in this study are included in the article/Supplementary Material. Further inquiries can be directed to the corresponding author.

## Supplementary Materials

The following supporting information can be downloaded at: https://www.mdpi.com/article/10.3390/foods14101816/s1, Table S1: Primer sequences used for RT-PCR experiments.
