# Supplementary material for: Therapeutic Mechanism of Kynurenine, a Metabolite of Probiotics, on Atopic Dermatitis in Mice
Source: Foods. 2025 May 20;14(10):1816. doi: 10.3390/foods14101816 (PMC12111421; doi:10.3390/foods14101816)
Supplement: Supplementary file 1 [file foods-14-01816-s001.zip › foods-3621249-supplementary.pdf]

foods

MDPI

Article

Therapeutic Mechanism of Kynurenine, a Metabolite of Probiotics, on Atopic Dermatitis in Mice

Yixuan Li †, Mingxin Li †, Qingyu Ren, Chunqing Ai, Shugang Li, Shouhao Zhao, Huan Li, Donglin Sui and Xiaomeng Ren *

Collaborative Innovation Center of Seafood Deep Processing, National Engineering Research Center of Seafood, School of Food Science and Technology, Dalian Polytechnic University, Dalian 116304, China; lyxqaq9527@163.com (Y.L.); 15998327512@163.com (M.L.); 19841133861@163.com (Q.R.); acqdongying@163.com (C.A.); lishugang688@163.com (S.L.); shouhao1105@163.com (S.Z.); lh276704997@163.com (H.L.); sdl3303@163.com (D.S.)

* Correspondence: rxmfighting@163.com

† These authors contributed equally to this work.

Table S1. Primer sequences used for RT-PCR experiments.

Genes	Forward Primer	Reverse Primer
ZO-1	GACCAATAGCTGATGTTGCCAGAG	TATGAAGGCGAATGATGCCAGA
Occludin	CTCGGTACAGCAGCAATGGT	TCATAGTGGTCAGGGTCCGT
Cauldin-2	TTCTTGAGGCTGCTTGTGG	GCTCTTGCTCCTTGAACACC
IL-5	CTCTGTTGACAAGCAATGAGACG	TCTTCAGTAT GTCTAGCCCCTG
IL-10	CTTACTGACTGGCATGAGGATCA	GCAGCTCTAGGAGCATGTGG
IL-13	CAGCCTCCCCGATACCAAAA	CTCCTCATTAGAAGGGGCCG
